# Development of improved method to identify and analyze lung fibrocytes with flow cytometry in a reporter mouse strain

**DOI:** 10.1002/iid3.361

**Published:** 2020-12-24

**Authors:** Hiroshi Kawano, Kazuya Koyama, Haruka Nishimura, Yuko Toyoda, Kozo Kagawa, Seidai Sato, Nobuhito Naito, Hisatsugu Goto, Yutaka Inagaki, Yasuhiko Nishioka

**Affiliations:** ^1^ Department of Respiratory Medicine & Rheumatology, Graduate School of Biomedical Sciences Tokushima University Tokushima Japan; ^2^ Department of Regenerative Medicine, Center for Matrix Biology and Medicine, Graduate School of Medicine Tokai University Isehara Japan

**Keywords:** CD11b, CD11c, fibrocyte, Gr‐1, pulmonary fibrosis

## Abstract

**Introduction:**

Fibrocytes are emerging myeloid‐derived circulating cells that can migrate into damaged tissues and usually contribute to their repair. Key features of fibrocytes include the expression myeloid markers, production of extracellular matrix proteins, and secretion of various humoral factors that activate resident fibroblasts; they also have the potential to differentiate into fibroblasts. However, no specific surface markers have been identified to identify fibrocytes in vivo. One reason could be that the method used to detect fibrocytes requires intracellular collagen staining.

**Methods:**

In the present study, to establish an improved method for the detection of lung fibrocytes and to analyze viable fibrocytes, we used collagen I(α)2‐green fluorescent protein (Col‐GFP) reporter mice, which had undergone the intratracheal instillation of bleomycin (BLM).

**Results:**

Using flow cytometry to gate out cells with autofluorescence, we clearly found that CD45^+^ GFP^+^ cells resided in the lungs of Col‐GFP mice at a steady state and these cells increased after BLM injury, peaking at Day 14. These cells expressed not only known cell surface markers of fibrocytes, but also some novel markers, in addition to a low level of collagen I in comparison to CD45^−^ GFP^+^ cells.

**Conclusion:**

Our findings suggest that the improved method can be a useful for the detection of pure lung fibrocytes and allows us to further analyze the characteristics of viable fibrocytes.

## Graphical abstract

In this report, we established improved method for detection of lung fibrocytes. Using flow cytometry to gate out cells with autofluorescence and combine expression pattern of CD11b, CD11c, Gr‐1, we clearly found fibrocytes resided in lungs.

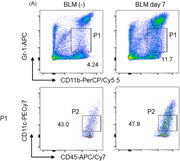

**Abbreviations**
BLM: bleomycin
GFP: green fluorescent protein
ILD: interstitial lung disease
IPF: idiopathic pulmonary fibrosis
MDSC: myeloid derived suppressor cell
MHC II: MHC class II

## 1 INTRODUCTION

Interstitial lung diseases (ILDs) are intractable problems that remain largely unknown and the priority to overcome these conditions is high. Idiopathic pulmonary fibrosis (IPF), the most severe form of ILD, has a poor prognosis, with a median survival time from the diagnosis ranging from 2.5 to 3.5 years, which is shorter than that in various malignant tumors.^1^ Recent reports have suggested that non‐IPF progressive fibrotic diseases, including chronic hypersensitivity pneumonitis, connective tissue disease‐related ILDs, and idiopathic nonspecific interstitial pneumonia, might have similar pathological phenotypes and clinical courses to IPF.^2^, ^3^ Thus, the development of more effective strategies to treat ILDs has become an urgent medical issue for pulmonologists.

Fibrocytes are myeloid‐derived circulating cells with characteristics of both fibroblasts and monocytes, which are described in 1994 by Bucala et al.^4^ Since this discovery, there have been an increasing number of reports on the characterization of fibrocytes and their role in the pathogenesis of various human diseases.^5^ Fibrocytes can migrate into damaged tissues and usually contribute to their repair.^6^, ^7^, ^8^ The key features of fibrocytes include the expression of myeloid markers such as CD34, CD45, CD14 and CD11a, CD11b, the production of extracellular matrix proteins such as collagen‐I, vimentin, and α‐smooth muscle actin, and the secretion of various humoral factors that activate resident fibroblasts; they also have the potential to differentiate into fibroblasts.^5^, ^9^

Because fibrocytes have unique phenotypes, as mentioned above, they are regarded as an important effector cell and treatment target in various ILDs. Indeed, a recent report demonstrated the treatment effect of serum amyloid P (Pentraxin 2), which can suppress the differentiation of fibrocytes, in phase II randomized clinical trial to IPF.^10^ However, specific surface markers to identify fibrocytes in vivo have not been identified. One reason could be that the method to detect fibrocytes requires intracellular collagen staining. The lack of specific surface markers is a major obstacle to the analysis of viable fibrocytes in vivo and the development of new drugs targeting fibrocytes. In the present study, to establish an improved method for the detection of lung fibrocytes and to analyze viable fibrocytes, we used collagen I(α)2‐green fluorescent protein (Col‐GFP) reporter mice, which had undergone intratracheal instillation of BLM. Using flow cytometry to gate out cells with autofluorescence, we clearly found CD45^+^ GFP^+^ cells residing in the lungs of Col‐GFP mice at a steady state and demonstrated that these cells increased after BLM injury. These cells expressed not only known cell surface markers of fibrocytes, but also some novel markers, in addition to a low level of collagen I in comparison to CD45^−^ GFP^+^ cells. Our findings suggest that the improved method can be useful for the detection of pure lung fibrocytes and allows us to further analyze the characteristics of viable fibrocytes.

## 2 MATERIALS AND METHODS

### 2.1 Mice

Collagen I(α)2‐EGFP reporter mice (Col‐GFP mice; C57BL/6 background) were generated as described previously.^11^ The mice were maintained in accordance with the Guidelines for Animal Experimentation of Tokushima University. Animal experiments were approved by the national and local authorities and conducted according to institutional and national guidelines. All experimental mice were eight to twelve weeks old and male.

### 2.2 Bleomycin exposure

Mice were anesthetized with the inhalation of 4% isoflurane and underwent the intratracheal instillation of 1 mg/kg of bleomycin sulfate (Tokyo Chemical Industry Co.) dissolved in saline as a single dose on Day 0.

### 2.3 Lung cell preparation and flow cytometry

In brief, lungs were isolated from mice and cut into small pieces. The fragments were digested in DMEM (Thermo Fisher Scientific) supplemented with 10% fetal bovine serum (FBS), 100 U/ml penicillin, 100 μg/ml streptomycin, 0.1% collagenase A (Roche)/0.1% dispase (Roche), and 10 U/ml DNase I (Roche) at 37°C for 60 min. The lung fragments were filtrated through a 70‐µm Cell Strainer (Greiner Bio‐One) into phosphate‐buffered saline, containing 5 mM ethylenediaminetetraacetic acid and 0.5% FBS. The single cell suspensions were divided into 1 × 10^6^ cells each per analysis and underwent a flow cytometric analysis with a FACS Versa (BD Biosciences‐Pharmingen). For the staining, fluorescence‐conjugated monoclonal antibodies against the following targets were used at a concentration of 0.25 µg/10^6^ cells in 100 µl volume: CD45 APC/Cy7 or PerCP/Cy5.5, CD11b PECy7 or PerCP/Cy5.5, CD11c PECy7, Gr‐1 PECy7 or APC, CD34 PECy7, CD40 PECy7, CD64 PECy7, CD80 PECy7, CD86 PECy7, IA/IE PECy7, F4/80 PECy7 or APC, CXCR4 biotin, Sca‐1 PECy7, Ly6C PECy7, Ly6G PECy7, MerTK PECy7, all from BioLegend, as were 7‐AAD Viability Staining Solution and PECy7‐conjugated Streptavidin. Each analysis of flow cytometry was performed through three experiments with a total of six mice per group.

### 2.4 RNA extraction and quantitative polymerase chain reaction (PCR) analysis

Sorted CD45^+^ empty PE^−^ CD11b^high^ CD11c^intermediate^ Gr‐1^intermediate^ GFP^+^ cells or CD45^−^ GFP^+^ cells from the lungs of BLM‐treated Col‐GFP mice were lysed in Buffer RLT (Qiagen) supplemented with 1% β‐mercaptoethanol (Sigma‐Aldrich). RNA was extracted using RNeasy Mini Kits (Qiagen) and made into complementary DNA (cDNA) with a SuperScript VILO cDNA Synthesis Kit (Thermo Fisher Scientific), in accordance with the manufacturer's instructions. qPCR analyses were performed using TB Green^Ⓡ^ Premix Ex Taq^TM^ II (TaKaRa) and run on a Real‐time PCR System (Applied Biosystems). Data were analyzed using the ΔΔ*C*_t_ method with Gapdh as housekeeping controls and the following primers: *Col1a1* (forward: 5ʹ‐GGTCCACAAGGTTTCCAAGG‐3ʹ, reverse: 5ʹ‐GCTGTTCCAGGCAATCCAC‐3ʹ), *Col1a2* (forward: 5ʹ‐GGAGGGAACGGTCCACGAT‐3ʹ, reverse: 5ʹ‐GAGTCCGCGTATCCACAA‐3ʹ), *GAPDH* (forward: 5ʹ‐GAAGGTGAAGGTCGGAGTC‐3ʹ, reverse: 5ʹ‐GAAGATGGTGATGGGATTTC‐3ʹ).

### 2.5 Purification of fibrocytes

Murine fibrocytes were isolated according to previously published methods.^12^ The lungs were harvested from BLM‐treated mice on Day 7 and minced with razor blades. The minced lungs were treated with 0.1% collagenase A (Roche)/0.1% dispase (Roche), and 10 U/ml DNase I (Roche) to produce single cell suspensions and then cultured in fibronectin‐coated 10‐cm dish for 7 days. Finally, adherent cells were harvested.

### 2.6 Statistical analysis

Data were analyzed using GraphPad Prism 8. All results are expressed as the mean ± SEM. The statistical analysis was performed using the Student two‐tailed unpaired *t* test for comparisons between two groups. *p* Values of <.05 were considered to indicate statistical significance.

## 3 RESULTS

### 3.1 Identification of myeloid‐derived cells with the GFP signal reporting collagen I(α)2 expression in the lungs of transgenic mice

**Figure 1:**
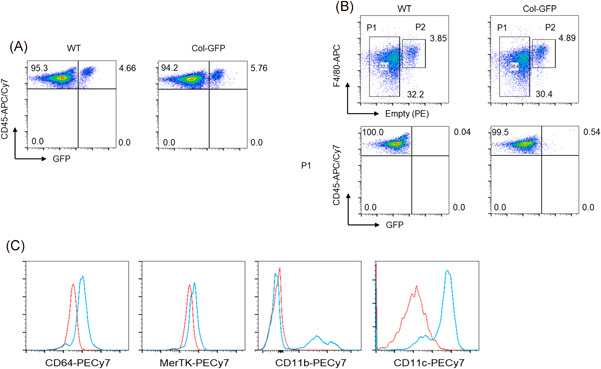
Detection of CD45^+^ GFP^+ ^cells in the lungs of the Col‐GFP reporter strain. (A) Total lung cells from adult wild‐type mice and the Col‐GFP reporter strain were isolated enzymatically and the expression of GFP together with CD45 (APC/Cy7) was evaluated by flow cytometry. (B) FACS profiles from CD45^+^ cells isolated from the same wild‐type mice and the Col‐GFP reporter strain are shown at the upper panel. After gating out F4/80 (APC)^+^ empty PE^+^ cells (P2 fraction), GFP^+^ cells (P1 fraction) were present in the Col‐GFP reporter strain (lower right). (C) The surface phenotype of gated out F4/80^+^ empty PE^+^ cells was examined by flow cytometry. Those cells were stained with CD64 PECy7, MerTK PECy7, CD11b PECy7, CD11c PECy7 antibodies or each isotype control antibody. Relative expression of each molecule is shown in histograms as blue lines overlaid with negative controls (red lines). One representative experiment from a total of three repeats with a total of six mice per group is shown. GFP, green fluorescent protein

To develop a better method for detecting fibrocytes, we utilized collagen I(α)2‐green fluorescent protein (Col‐GFP) reporter mice.^11^ We first investigated the GFP expression in CD45^+^ lung cells of adult Col‐GFP reporter mice using flow cytometry (Figure S1A). Unexpectedly, CD45^+^ GFP^+ ^cells were detected in both wild‐type and Col‐GFP mice (Figure 1A). We suspected that the GFP signal in wild‐type mice was a reflection of the autofluorescence from alveolar macrophages.^13^ Indeed, after gating out F4/80^+^ empty PE^+^ cells with autofluorescence, a few CD45^+^ GFP^+^ cells were only detectable in Col‐GFP reporter mice (Figures 1B and S1B). Gated out cells were CD64^+^ MerTK^+^ CD11b^−^ CD11c^+^, suggesting that these cells were consistent with macrophages (Figure 1C). Thus, using flow cytometry to gate out cells with autofluorescence, we clearly found that CD45^+^ GFP^+^ cells resided in the lungs of Col‐GFP reporter mice.

### 3.2 Bleomycin induced lung injury resulted in an increase of CD45^+^ GFP^+^cells that expressed known fibrocyte cell surface markers

**Figure 2:**
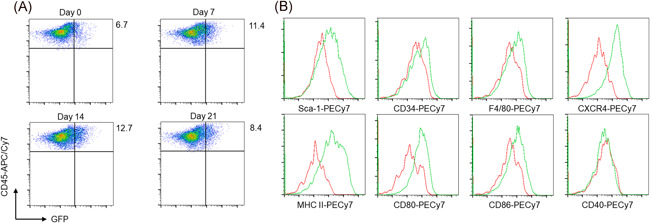
The surface phenotype of CD45^+^ GFP^+ ^cells in the lungs of the BLM‐treated Col‐GFP reporter strain. (A) The increase of CD45^+^ GFP^+^ lung cells from the Col‐GFP reporter strain upon treatment with BLM was monitored at 7 (upper right), 14 (lower left), and 21 days (lower right) after intratracheal instillation. (B) The surface phenotype of CD45^+^ GFP^+^ cells on Day 7 after BLM treatment was examined by flow cytometry. Relative expression of each molecule is shown in the histogram as green lines overlaid with negative controls (red lines). One representative experiment from a total of three repeats with a total of six mice per group is shown. BLM, bleomycin; GFP, green fluorescent protein

To elucidate phenotypes, the cell surface marker expression of CD45^+^ GFP^+^ cells was examined. Based on the fact that fibrocytes are recruited to injured tissues and contribute to their repair,^6^, ^7^, ^8^ Col‐GFP reporter mice were treated with BLM to collect large numbers of CD45^+^ GFP^+^ cells for flow cytometry. After the intratracheal instillation of BLM, the number of CD45^+^ GFP^+^ cells in the lungs peaked on Day 14 (Figure 2A), consistent with previous reports on fibrocyte recruitment.^14^ Next, the surface phenotype of CD45^+^ GFP^+^ cells on day 7 after BLM treatment was evaluated, as it was assumed that these cells had just recruited to the lungs. CD45^+^ GFP^+^ cells on day 7 expressed not only stem cell markers, such as CD34 and Sca‐1, but also some macrophage markers, including antigen presentation‐related molecules (Figure 2B). All of those have been reported to be expressed on fibrocytes.^15^ Thus, the results indicate that CD45^+^ GFP^+^ cells may contain fibrocytes.

### 3.3 CD45^+^ GFP^+^ cells show characteristic expression patterns of cell adhesion molecules

**Figure 3:**
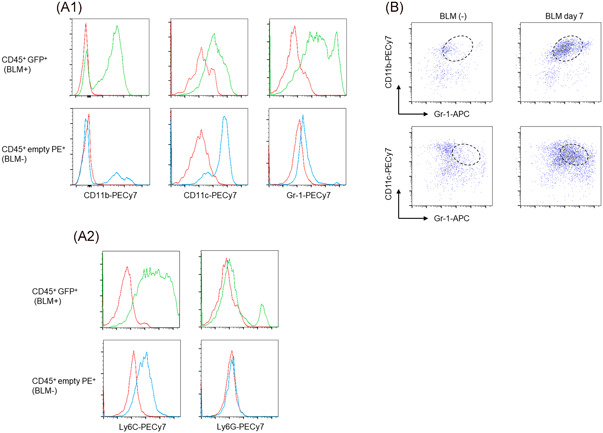
The characteristic expression pattern of cell adhesion molecules in CD45^+^ GFP^+^ cells. (A) The expression profiles of CD11b, CD11c, Gr‐1, Ly6C, and Ly6G in CD45^+^ GFP^+^ cells on Day 7 after BLM treatment (upper panels) were compared with CD45^+^ empty PE^+^ cells without BLM treatment (lower panels) by flow cytometry. (B) FACS profiles from CD45^+^ GFP^+^ cells isolated from the Col‐GFP reporter strain with (right panels, on Day 7) or without BLM treatment (left panels) are shown. One representative experiment from a total of three repeats with a total of six mice is shown. BLM, bleomycin; GFP, green fluorescent protein

Fibrocytes express numerous molecules involved in cell adhesion and cell‐cell interactions that play an important role in their recruitment to injured tissues.^16^ We therefore focused on the cell adhesion molecules on CD45^+^ GFP^+^ cells and found that these cells showed the characteristic expression pattern of CD11b, CD11c, and Gr‐1, in comparison to CD45^+^ empty PE^+^ macrophages without BLM treatment (Figure 3A). Namely, after BLM injury, most CD45^+^ GFP^+^ cells showed the following expression pattern: CD11b^high^, CD11c^intermediate^, and Gr‐1^intermediate^ (Figure 3B). This suggests that fibrocytes could be included within a population of CD45^+^ empty PE^−^ GFP^+^ CD11b^high^ CD11c^intermediate^ Gr‐1^intermediate^ cells. Gr‐1‐specific antibodies bind to two different epitopes: Ly6G and Ly6C. CD45^+^ empty PE^−^ GFP^+^ cells showed a more evident expression of Ly6C than Ly6G (Figure 3A).

### 3.4 Flow cytometry using the combined expression pattern of CD11b, CD11c, Gr‐1 enables the detection of more purified lung fibrocytes

**Figure 4:**
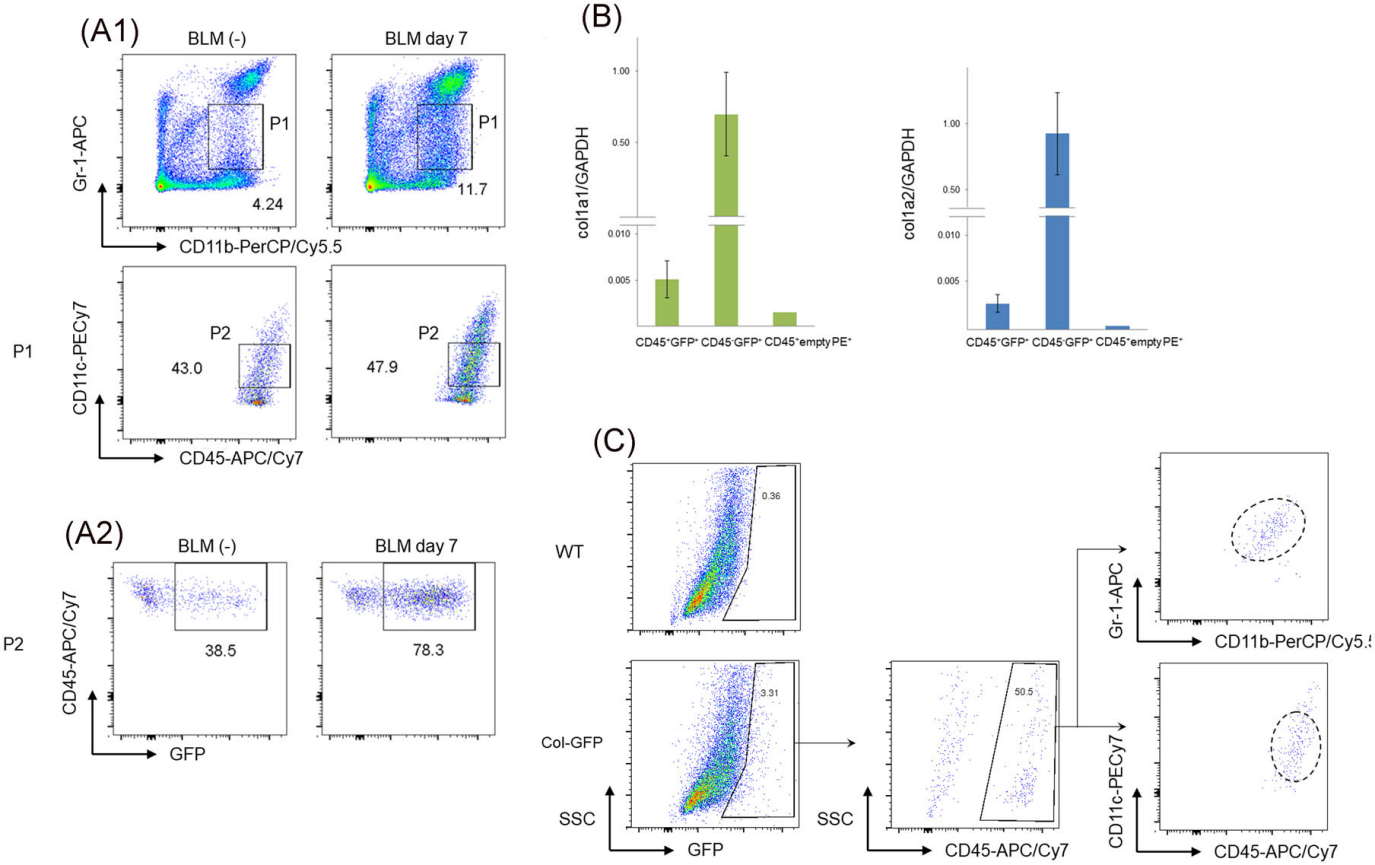
Validation of the method of sorting CD45^+^empty PE^−^ CD11b^high^CD11c^intermediate^Gr‐1^intermediate^cells by flow cytometry. (A) The gating strategy for the collection of more purified CD45^+^ GFP^+^ cells. Approximately 80% of CD45^+^ empty PE^−^ CD11b^high^ CD11c^intermediate^ Gr‐1^intermediate^ cells were GFP^+^ in the Col‐GFP reporter strain on day 7 after BLM treatment (bottom right panel). One representative experiment from a total of three repeats with a total of six mice is shown. (B) The expression of collagen I(α)1 and collagen I(α)2 in sorted CD45^+^ empty PE^−^ CD11b^high^ CD11c^intermediate^ Gr‐1^intermediate^ GFP^+^cells from the lungs were examined by RT‐PCR. (C) The phenotype of fibrocytes in the lungs of the Col‐GFP reporter strain treated with BLM. The lungs treated with BLM were harvested on Day 7. Single cell suspensions were collected from the minced lungs. The cells were then cultured on dishes and adherent cells were harvested. Flow cytometry was performed with GFP^+^ adherent cells using the gating strategy shown in (A). One representative experiment from a total of three repeats with a total of six mice is shown. BLM, bleomycin; GFP, green fluorescent protein; RT‐PCR, reverse‐transcription polymerase chain reaction

Based on the characteristic expression pattern of CD11b, CD11c, Gr‐1 in CD45^+^ GFP^+^ cells, the validity of a flow cytometry method using these markers to identify fibrocytes was verified. Notably, upon the detection of CD45^+^ empty PE^−^ CD11b^high^ CD11c^intermediate^ Gr‐1^intermediate^ lung cells from BLM‐treated Col‐GFP reporter mice, we found that approximately 80% of the cells were GFP^+^ (Figure 4A). These cells expressed known cell surface markers of fibrocytes in addition to a low level of collagen I in comparison with CD45^−^ GFP^+^ cells (Figure 4B). Moreover, murine fibrocytes isolated according to a previously published method^12^ showed exactly the same expression pattern on CD11b, CD11c, and Gr‐1, further confirming that our improved flow cytometry method could achieve the collection of greater numbers of purified fibrocytes (Figure 4C).

## 4 DISCUSSION

In this study, we clearly found that fibrocytes resided in the lungs of Col‐GFP reporter mice using an improved method of flow cytometry to gate out cells with autofluorescence (empty PE^+^ cells), followed by sorting CD11b^high^ CD11c^intermediate^ Gr‐1^intermediate^ cells. These cells expressed known fibrocyte cell surface markers, in addition to a low level of collagen I in comparison to CD45^−^ GFP^+^ cells, which would contain fibroblasts.

In previous studies, fibrocytes have been identified as spindle‐shaped cells that meet the minimum definition criteria, including the detection of collagen production and the surface expression of CD45 and/or CD34.^5^, ^9^ In spite of insufficiency, these criteria still apply to this day, as there are still no specific markers for fibrocytes. Various cell surface markers used for the identification of fibrocytes are commonly expressed on other hematopoietic cells, especially macrophages.^5^, ^9^, ^15^ As a result, the methods to detect fibrocytes have required intracellular collagen staining, making it difficult to purify and analyze fibrocytes as viable cells.

Given that CD45^+^ GFP^+^ cells are theoretically defined as fibrocytes in Col‐GFP reporter mice, we considered the collection of these cells to be a useful method to identify lung fibrocytes. However, proving the hypothesis required some elaboration, including gating out empty PE^+^ cells from CD45^+^ lung cells. Alveolar macrophages have been reported to emit high autofluorescence due to their vigorous phagocytosis of diverse materials.^13^ Indeed, gated out cells were consistent with the phenotypes of alveolar macrophages. Thus, the negative selection of empty PE^+^ cells could contribute to raising the purity of fibrocyte collection.

Another contrivance was the use of the combination of three common adhesion molecules (CD11b, CD11c, Gr‐1) as recognition markers for fibrocytes. Like macrophages, fibrocytes express the adhesion proteins CD11b, CD11c, and CD11d.^12^ On the other hand, Gr‐1 is a glycosylphosphatidylinositol (GPI)‐linked protein, also known as Ly‐6G/Ly‐6C, and expressed not only on granulocytes and macrophages but also transiently on immature bone marrow cells in the monocyte lineage.^17^ Although there have been no reports on the Gr‐1 expression on fibrocytes, we found that most CD45^+^ empty PE^−^ GFP^+^ cells in Col‐GFP reporter mice were CD11b^high^ CD11c^intermediate^ Gr‐1^intermediate^ after BLM treatment, leading to the development of the improved method of flow cytometry that could further raise the purity for fibrocyte collection.

Considering the expression patterns, it is possible that CD45^+^ empty PE^− ^CD11b^high^ CD11c^intermediate^ Gr‐1^intermediate^ cells may also include myeloid‐derived suppressor cells (MDSCs). MDSCs are a heterogeneous population of cells, composed of precursors of macrophages, granulocytes, dendritic cells, and myeloid cells at different stages of differentiation. MDSCs,^18^ characterized by the co‐expression of Gr‐1 and CD11b in mice, have been shown to inhibit T cell activation in different tumor models. Several studies have already established the existence of distinct mononuclear and polynuclear subpopulations based on the expression of Ly6G and Ly6C, both of which are detected by Gr‐1‐specific antibodies. CD11b^+^ Ly6G^+^ Ly6C^low^ MDSC have been described to have a more granulocytic phenotype, whereas CD11b^+^ Ly6G^−^ Ly6C^high^ cells display a more monocytic phenotype.^18^ In our experiments, CD45^+^ GFP^+^ cells were CD11b^+^ Gr‐1^intermediate^ with the expression of the collagen I gene, and we interpreted that these cells were discriminated from MDSCs.

In addition, several new cell populations with similar phenotypes to our CD45^+^ GFP^+^ cells have been reported. Satoh et al.^19^ demonstrated an atypical monocyte committed progenitor involved in fibrosis. They showed that Ceacam1^+^ Msr1^+^ Ly6C^−^ F4/80^−^ Mac1^+^ monocytes, termed segregated‐nucleus‐containing atypical monocytes (SatM) were critical for fibrosis. Our CD45^+^ GFP^+^ cells were Ly6C^+^, suggesting that these cells are different from SatM.^19^ Sendo et al.^20^ reported that CD11b^+^ Gr‐1^dim^ tolerogenic dendritic cell‐like cells were expanded in ILD in an inflammatory arthritis model. Most CD11b^+^ Gr‐1^dim^ tolerogenic dendritic cell‐like cells were F4/80^−^ and CD11c^+^ and were also dissimilar to our CD45^+^ GFP^+^ cells.^20^

The present study was associated with some limitations. CD45^+^ GFP^+^ cells in the lungs of Col‐GFP reporter mice can be considered to consist of at least three types of cells: macrophages with autofluorescence, some phagocytes incorporating GFP, and myeloid derived cells expressing collagen I, such as fibrocytes. We gated out auto‐fluorescent cells and showed that CD45^+^ GFP^+^cells sorted by our method express the collagen I gene, suggesting that these cells actually meet the definition of fibrocytes. However, the collagen I gene expression level was lower than expected. For that reason, it is possible that the expression levels of endogenous gene reported by transgene may vary by using the same promoter, and/or that CD45^+^ GFP^+^ cells contain a large portion of phagocytes incorporating GFP. Another possibility is the existence of CD45^−^ GFP^+^fibrocytes in our model. Fibrocytes are reported to lose the expression of hematopoietic surface markers such as CD45 during differentiation and can become indistinguishable from resident fibroblasts.^6^, ^15^ The recent study has demonstrated the existence of CD45^−^ CD11b^−^ cells that are hematopoietic origin with distinct characteristics of fibrocytes in a cutaneous healing model using the ingeniously designed transgenic mice.^21^ We assume that CD45^−^ CD11b^−^ Col1 (GFP)^+^ hematopoietic‐derived cells might exist in BLM‐injured lungs of our reporter strain as well. However, a definitive sorting of those cells would be impossible without the use of the double transgenic (Vav‐Cre and mTmG) strain model.^21^ Our study lacks some functional assays in vivo, for example, the assessment of the profibrotic effects of transferring CD45^+^ GFP^+^ cells sorted by our method from the reporter strain to wild‐type mice. Bone marrow cell transfer experiments should also be performed to confirm the usefulness of our method.

In conclusion, our findings suggest that the improved method can be useful for the detection of pure lung fibrocytes and allows us to further analyze the characteristics of viable fibrocytes. Further studies, using approaches such as a DNA microarray or single cell RNA‐seq analysis of these cells, would expand the possibilities to realize the detection of more specific markers of fibrocytes.

## CONFLICT OF INTERESTS

The authors declare that there are no conflict of interests.

## AUTHORS CONTRIBUTION

Hiroshi Kawano contributed to design the study, carried out data analysis, and wrote the manuscript. Kazuya Koyama helped for flow cytometry and its quantification. Haruka Nishimura, Yuko Toyoda, Kozo Kagawa, Seidai Sato, and Hisatsugu Goto contributed to design the study and assess the data. Nobuhito Naito helped for RT‐PCR. Yutaka Inagaki helped for handling Col‐GFP mice and reviewed the manuscript. Yasuhiko Nishioka contributed to design the study and reviewed the data and manuscript.

## Supporting information

Supporting information.

Supporting information.

Supporting information.

## Data Availability

The data that support the findings of this study are available from the corresponding author upon reasonable request.
