# Supplementary figures and images for: Development of improved method to identify and analyze lung fibrocytes with flow cytometry in a reporter mouse strain
Source: Immun Inflamm Dis. 2020 Dec 24;9(1):120–7. doi: 10.1002/iid3.361 (PMC7860606; doi:10.1002/iid3.361)

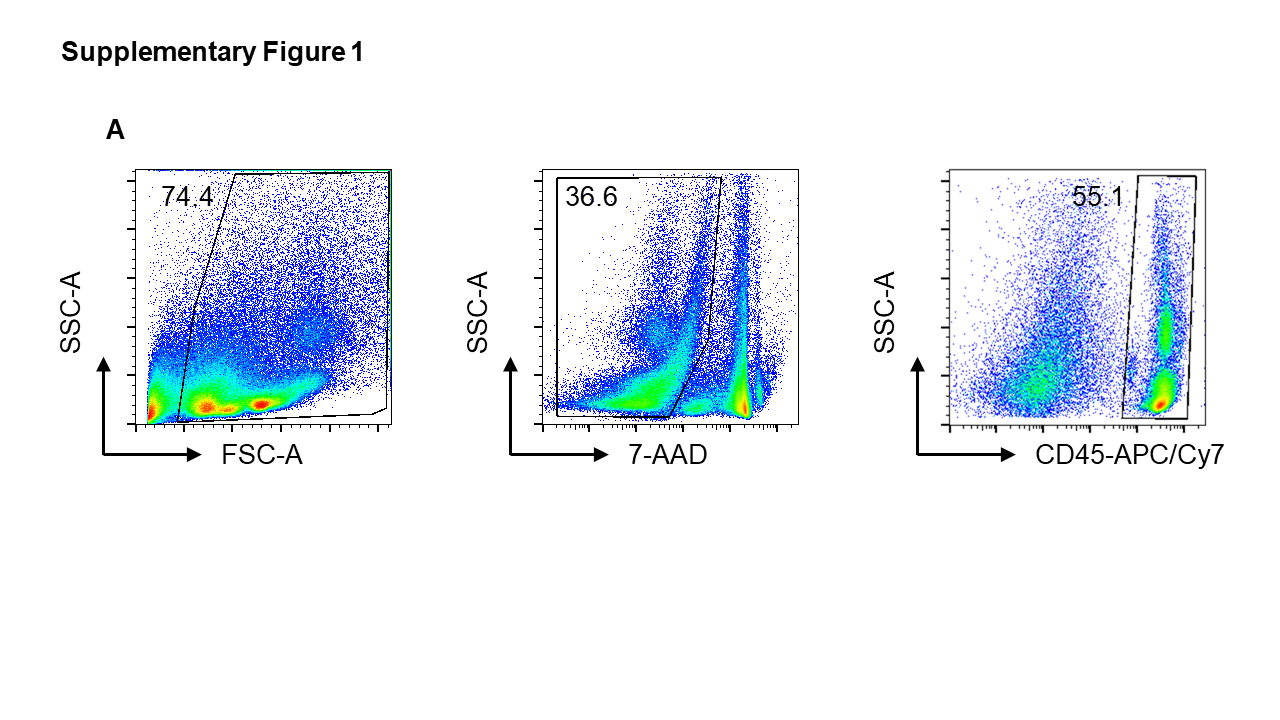

Supplement: Supplementary file 1 — Supporting information. [file IID3-9-120-s001.tif]

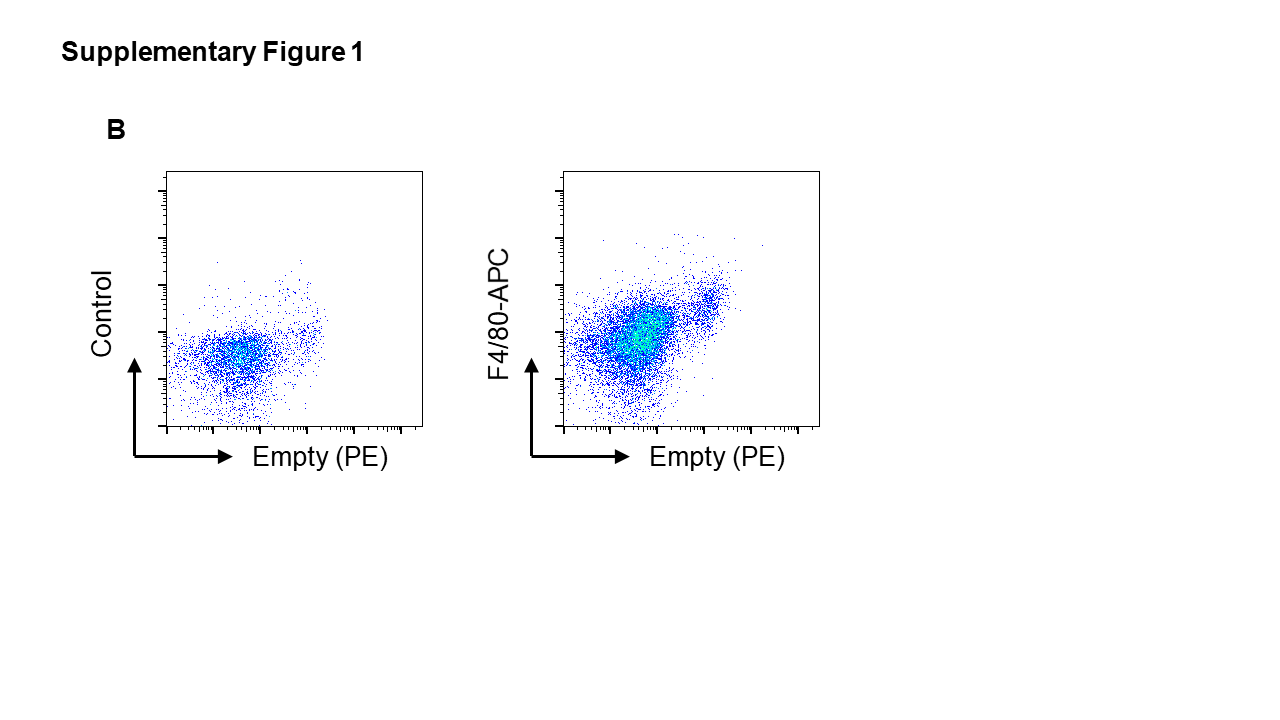

Supplement: Supplementary file 2 — Supporting information. [file IID3-9-120-s002.tif]
