# Supplementary material for: Development of improved method to identify and analyze lung fibrocytes with flow cytometry in a reporter mouse strain
Source: Immun Inflamm Dis. 2020 Dec 24;9(1):120–7. doi: 10.1002/iid3.361 (PMC7860606; doi:10.1002/iid3.361)
Supplement: Supplementary file 3 — Supporting information. [file IID3-9-120-s003.docx]

**Supporting information**

**Supplementary Figure 1 | Demonstration of the gating strategy for the flow cytometric analysis of mouse lung cells.**

A single cell suspension was prepared from the lungs of a wild type or a Col-GFP reporter C57BL/6 mouse and stained with fluorescence-conjugated monoclonal antibodies against CD45 (APC/Cy7), F4/80 (APC) and 7-AAD. A: Lung cells without cellular debris were identified by their scatter properties (FSC-A x SSC-A plot) and then dead cells were excluded by gating on 7-AAD^-^ cell population. The surface CD45^+^ cell population represents hematopoietic cells. B: CD45^+^ cells included F4/80^+^ empty PE^+^ cell population.

Representative plots of a BLM-untreated mouse are shown.
